# Large-scale cortical functional networks are organized in structured cycles

**DOI:** 10.1038/s41593-025-02052-8

**Published:** 2025-08-27

**Authors:** Mats W. J. van Es, Cameron Higgins, Chetan Gohil, Andrew J. Quinn, Diego Vidaurre, Mark W. Woolrich

**Affiliations:** 1 Oxford Centre for Human Brain Activity (OHBA), Oxford Centre for Integrative Neuroimaging, Department of Psychiatry, University of Oxford, Oxford, UK; 2 Resonait Medical Technologies Pty Ltd, Sydney, New South Wales Australia; 3 Brain and Mind Centre, University of Sydney, Sydney, New South Wales Australia; 4 School of Computer Science, University of Sydney, Sydney, New South Wales Australia; 5 Centre for Human Brain Health, School of Psychology, University of Birmingham, Birmingham, UK; 6 Center for Functionally Integrative Neuroscience, Department of Clinical Medicine, Aarhus University, Aarhus, Denmark

**Keywords:** Sensory processing, Dynamic networks, Stochastic networks, Network models, Attention

## Abstract

The brain seamlessly performs a diverse set of cognitive functions like attention, memory and sensory processing, yet it is unclear how it ensures that each of these is fulfilled within a reasonable period. One way in which this requirement can be met is if each of these cognitive functions occurs as part of a repeated cycle. Here we studied the temporal evolution of canonical, large-scale, cortical functional networks that are thought to underlie cognition. We showed that, although network dynamics are stochastic, the overall ordering of their activity forms a robust cyclical pattern. This cyclical structure groups states with similar function and spectral content at specific phases of the cycle and occurs at timescales of 300–1,000 ms. These results are reproduced in five large magnetoencephalography datasets. Moreover, we show that metrics that characterize the cycle strength and speed are heritable and relate to age, cognition and behavioral performance. These results show that the activations of a canonical set of large-scale cortical functional networks are organized in an inherently cyclical manner, ensuring periodic activation of essential cognitive functions.

## Abstract (web summary)

The human brain cycles through a repertoire of brain networks on a 1-second timescale during rest and tasks. This cycling appears to allow periodic engagement of essential cognitive functions, with the speed of cycling linked to genetics and age.

## Main

The human brain fulfills many cognitive and homeostatic functions in a flexible and adaptive manner, which is essential for survival. Yet, it is unclear how it is organized to ensure that each of these is within a certain time frame when the brain is in a nonstructured temporal environment. One way in which this requirement can be met is if each of the cognitive functions occurs as part of a repeating cycle. As large-scale cortical networks, studied through functional brain imaging, are thought to underlie specialized cognitive functions^1–10^, we can examine the dynamics of these cortical networks to see whether cyclical patterns exist.

Research into spontaneous brain activity recorded in wakeful rest using magnetoencephalography (MEG), electroencephalography^1,11–13^ and functional magnetic resonance imaging (fMRI)^14–16^ has shown that transitions between cortical networks in wakeful rest, or resting state, networks are nonrandom and different levels of organization have been observed. For example, multimodal evidence from MEG^11,17^ and fMRI^5,18^ suggests that the default mode network (DMN) and the dorsal attention network (DAN), associated with an inward versus an outward orientation of attention, respectively, are anti-correlated and unlikely to transition into each other directly. Moreover, recent results from fMRI show that the nonrandom transitions between resting state networks contain a hierarchical component, with clusters of brain states that are more likely to transition into each other within but not across clusters^14^. These asymmetries in transition probabilities between brain networks have further been shown to be more directional in states of higher awareness and in more physically and cognitively demanding tasks in both electrophysiology^19–21^ and fMRI^22,23^. However, the existence of cyclical patterns between a full set of canonical, large-scale cortical networks has not previously been shown.

Here we investigated the temporal dynamics of large-scale cortical networks in multiple MEG datasets obtained during wakeful rest. We developed a new method for quantifying the transition asymmetries of these networks at a range of timescales, which showed that asymmetrical transitions are ubiquitous in human brain activity. Moreover, although individual transitions were stochastic, together they produced a robust cyclical pattern of cortical network activations on timescales of 300–1,000 ms, an order of magnitude longer than the average lifetime of a single network. These patterns were reproduced in five independent datasets and robustly showed a preferred position of each brain network in the cycle. Furthermore, we showed that cyclical summary metrics are heritable and relate to age, cognition and behavioral performance. Together, these results show an overarching flow of cortical networks, suggesting that cortical network activations are inherently cyclical, ensuring periodic activation of essential cognitive functions.

## Results

### Functional brain networks activate in cycles

To explore the temporal dynamics of large-scale functional brain networks in resting state MEG, we first conducted a secondary analysis of previously published results^24^. This new analysis considered the longer-term patterns of resting state network (RSN) activity in the Nottingham MEG UK dataset (55 participants)^25^. Using hidden Markov modeling (HMM), the analysis (Methods) identified *K* = 12 states, reflecting distinct brain networks with unique spatial configurations of power and coherence that reoccur at different points in time. States are inferred that best explain the multivariate distribution of activity across the entire brain whenever that state is active; they do not model any single spatial region in isolation, although spatially confined activity may nevertheless be characteristic of a particular state. The state descriptions of all network states are shown in Supplementary Fig. 1.

**Fig. 1:**
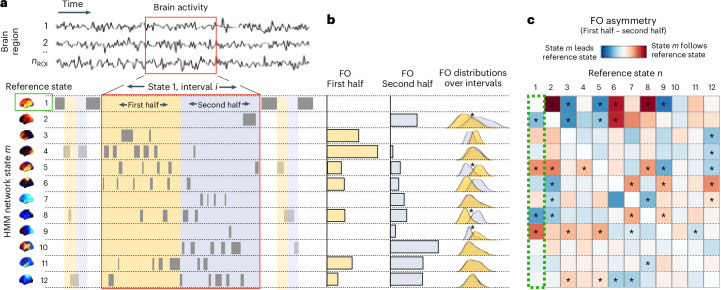
Schematic of TINDA, with state 1 as an example reference state. **a**, A segment of the (1 to *n* regions of interest (*n*_ROI_)) multi-region, resting state MEG data (top) and the inferred HMM state activations (bottom). Segment *i* is the period between reactivations of network state 1, which is further subdivided into two interval halves (first half yellow, second half blue). **b**, The FO (that is, relative time spent) for each network state in both intervals in **a** (left) and the FO distributions over all state-1-to-state-1 intervals (right). Asterisks represent statistical significance (*P* < 0.05, Bonferroni-corrected). **c**, The FO asymmetry matrix showing the mean FO difference over intervals between the two interval halves with respect to a reference state (in this example, state 1). This procedure is repeated for all reference states *n* to create the full FO asymmetry matrix, which is used in the results going forward. Asterisks denote significant (*P* < 0.05, Bonferroni-corrected) FO asymmetries. Note that each state gets used in turn as the reference state, with the outputs then combined within the TINDA procedure. The results from **a** and **b** are condensed in the column enclosed by the green dotted line. Source data

We characterized the tendency of network states to follow or precede each other using a new method called temporal interval network density analysis (TINDA; Fig. 1). This method focuses on the variable-length intervals between network state occurrences, which relaxes more common assumptions of fixed-length timing patterns, an approach that we show to be crucial to its success (Supplementary Fig. 4). For each reference state *n*, TINDA takes all intervals between reoccurrences of the same state (that is, state-*n*-to-*n* intervals) and partitions them evenly in half. It then defines the fractional occupancy (FO) asymmetry as the difference between the probability of another network state *m* occurring in the first half versus the second half of those intervals. This measure captures whether there is a tendency for a network state to follow, or precede, another state over variable timescales (Methods and Fig. 1d,e).

We used this method to investigate whether an overarching pattern emerged when every state’s tendency to follow or precede every other state was analyzed. To illustrate its use more clearly, we first used this method on the intervals defined by subsequent visits to state *n* = 1 (Fig. 1). This revealed that certain network states (state 5, *t*(54) = 5.1, *P* = 4.1 × 10^−6^, and state 9, *t*(54) = 6.4, *P* = 3.7 × 10^−8^) tend to occur after state 1, whereas other states (state 2, *t*(54) = −4.6, *P* = 2.3 × 10^−5^, and state 8, *t*(54) = −6.1, *P* = 9.9 × 10^−8^) tend to occur before state 1. All other states (3, 6, 7, 10, 11 and 12) did not exhibit significant asymmetrical activation probability after Bonferroni’s correction for multiple comparisons. In the interest of reproducibility, we repeated the same analysis for the equivalent state in two other large datasets (Cambridge Centre for Aging Neuroscience (Cam-CAN; *n* = 612)^26,27^ and the Human Connectome Project (HCP; *n* = 79)^28^) and found consistent results (Supplementary Fig. 2).

We next investigated whether asymmetries in activation probabilities also existed for other network states. Using TINDA on all pairs of network states, we confirmed that this was indeed the case and, moreover, that these pathways were unique to each state (Fig. 1c and Supplementary Fig. 2). All results that follow rely on the full FO asymmetry matrix (Fig. 1c), that is, where TINDA is applied to state-*n*-to-state-*n* intervals for $n\in 1:K$.

**Fig. 2:**
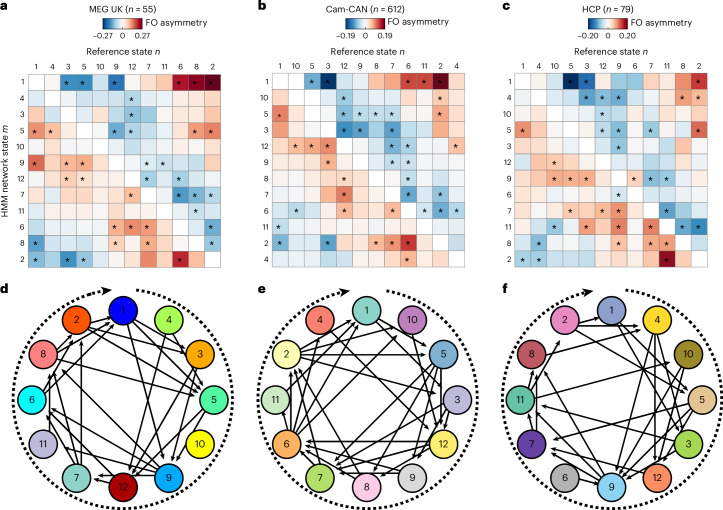
TINDA reveals cyclical activation of functional brain networks in three large MEG datasets. **a**–**c**, The group-average FO asymmetry in three large MEG datasets (MEG UK, *n* = 55 (**a**); Cam-CAN, *n* = 612 (**b**); HCP, *n* = 79 (**c**)), showing the activation probability of one network state (*y* axis) relative to another (*x* axis). Asterisks denote statistically significant elements (Bonferroni-corrected at α = 0.05, α = 10^−20^, α = 0.01, respectively). **d**–**f**, Visualizing the statistically significant FO asymmetries in the MEG UK (**d**), Cam-CAN (**e**), and HCP (**f**) datasets as directed edges reveal an overarching cyclical activation structure of functional brain networks. The colors of nodes in the different datasets are distinct to indicate that network state descriptions are inferred independently from each dataset. State numbers in Cam-CAN or HCP are matched to the MEG UK dataset (Methods), which is numbered in order of decreasing coherence. Source data

We then explored the possibility that the asymmetries in network activation probabilities are unified by an overarching structure. In particular, visual inspection of these networks raised the possibility that they were unified by a globally cyclical structure (Fig. 2d and Supplementary Video 1), an emergent dynamic that could not arise trivially from the first-order state asymmetries (*P* < 0.01; Supplementary Information Section II). We defined the cycle strength (*S*) to test the potential cyclical structure statistically (see Methods for details). Cycle strength is +1 for graphs where all transitions are perfectly clockwise, zero for completely stochastic graphs and negative for overall counterclockwise transitions (note that, when states are ordered to maximize *S*, negative cycle strength can be true only for individual participants, not for the group average, and vice versa when *S* is minimized). We confirmed that the cyclical pattern, as a result of all FO asymmetries together, could not have arisen by chance by permuting network state labels within each participant. In each of 1,000 permutations, the order of states was shuffled independently for each participant and cycle strength was computed using the optimal cycle order for that permutation; the observed cycle strength was significantly greater than in permutations (mean (s.d.): *S* = 0.066 (0.041), *P* < 0.001). Moreover, in additional control analyses, we ruled out the possibility that the cyclical pattern could arise from common (rhythmic) physiological artifacts; Supplementary Information Section VI.

In the interests of reproducibility, we replicated these analyses in the two other datasets, confirming both the presence of cyclical dynamics and the consistency of individual state ordering within the cyclical configuration across all datasets. HMMs were independently trained on each dataset, after which states were reordered to match the ordering in the MEG UK dataset (Methods); state numbers across the three datasets thus refer to equivalent network states. We confirmed that cycle strength was higher than permutations in both the Cam-CAN (*S* = 0.049 (0.033), *P* < 0.001; Fig. 2b,e) and the HCP (*S* = 0.048 (0.035), *P* < 0.001; Fig. 2c,f). This confirmed the presence of a cyclical structure in all three datasets, but it remained plausible that these were different cyclical structures. As we identified equivalent states in all three datasets, we could test whether the order of states in the cycle was comparable between datasets. We computed the cycle phase difference between equivalent states in each dataset and compared this to a random placement of states across the cycle (that is, permuting state positions 10,000×). This analysis confirmed that the order of states in the Cam-CAN cycle matched the order in MEG UK: the mean phase difference (Δ*θ*) between equivalent states was smaller than expected by chance (Δ*θ* = 0.645 rad, *P* = 0.0038; Supplementary Information Section III). Despite the use of an entirely different parcellation in HCP, the same was true in this dataset (Δ*θ* = 0.472 rad, *P* = 0.0001). These analyses thus show that the same cyclical dynamics can be observed across three independent datasets.

### Cycles are strongest over timescales of seconds

Given the strength of this cyclical activation pattern, we considered why it had not been characterized previously in the literature. TINDA differs from other methods of characterizing dynamics in that it measures dynamics over interstate intervals (ISIs) of variable length. These intervals have a highly dispersive distribution with a very long tail^11,24,29^. Common means of modeling temporal dynamics typically assume either a Markovian structure, as in our work (that is, that the state at one time point is conditionally dependent on only the immediately prior state^11^), or a structure of temporal dependency with fixed-length time lags^30^. Simulations from either of these models trained on the existing dataset illustrate why such a cyclical activation pattern would not have been detected in previous work without an additional post hoc analysis such as TINDA to capture dependencies that are not reflected explicitly in the model parameters. These simulations capture only a small part of this inherent cyclical structure, most of which is lost due to the variability of ISI durations (Supplementary Fig. 4). The fact that other models capture only a small part of this inherent cyclical structure underlines the importance of our new approach.

This also suggests two key temporal features of the cyclical patterns that we have characterized: first, that these cyclical patterns are instantiated over longer timescales and, second, that they do not have a regular cyclical period (Supplementary Information Section VI and Supplementary Video 1). To verify this quantitatively, we looked at the dependency of the FO asymmetry and cycle strength on the interval duration (that is, *T*^*m,i*^ with *m* = 1 in Fig. 1, the interval time between subsequent visits to the network state of interest (*m*)). We expected that, if cyclical patterns were instantiated over longer timescales, then the FO asymmetries and the characteristic cycle would be apparent only at longer interval times.

**Fig. 3:**
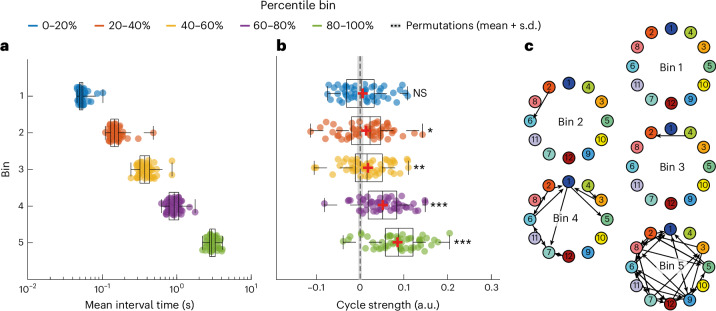
The observed cyclical organization of network state activations is driven by longer interval times in the MEG UK dataset (*n* = 55). For each participant and state, intervals were binned by interval times into five percentile bins. **a**, The mean duration of interval times pooled over all 12 states within each percentile bin. **b**, The cycle strength resulting from running TINDA on each percentile bin in **a**. Circles are individual participants, boxplots display the median, mean (+ s.d.), and 25th and 75th percentiles and whiskers indicate the minimal and maximal value in the group. The line and error bar around zero cycle strength are the mean and s.d. of the empirical permutation distribution based on 1,000 permutations. Significant cycle strength (permutation test, one sided) was observed for percentile bin 2 (*S* = 0.014, *P* = 0.025), bin 3 (*S* = 0.018, *P* = 0.0060), bin 4 (*S* = 0.052, *P* < 0.001) and bin 5 (*S* = 0.086, *P* < 0.001), but not for percentile bin 1 (*S* = 0.0060, *P* = 0.17). Significance is denoted by asterisks: ^*^P < 0.05, ^**^P < 0.01, ^***^P < 0.001 (Bonferroni-corrected). **c**, Graphs similar to Fig. 2d for binned interval times with increasing duration from top to bottom. Note that arrows are only shown for significant FO asymmetries, that is, bin 1 does not contain any significant asymmetries and is therefore empty. NS, not significant. Source data

To do this we partitioned the distribution of interval times (Fig. 3a) into five equally sized bins. We did this separately for each state to ensure that there was no state bias in each bin. This procedure resulted in each bin containing an average (s.d.) of 885 (111) intervals for each participant. We then reran the TINDA procedure separately on (the intervals from) each bin. Figure 3b shows that group mean cycle strength is close to zero for the bins with the shortest duration intervals and increases for bins with higher interval durations. Cycle strength is significantly higher than in permutations (Methods) in bins 2–5 and strongest in the bin containing the longest interval durations (with a mean interval time of 3 s). This was replicated in the Cam-CAN and HCP datasets (Supplementary Fig. 5) and together these results prove that the cyclic activation of network states is occurring at timescales of the order of seconds.

### Cycles connect networks with similar properties and function

**Fig. 4:**
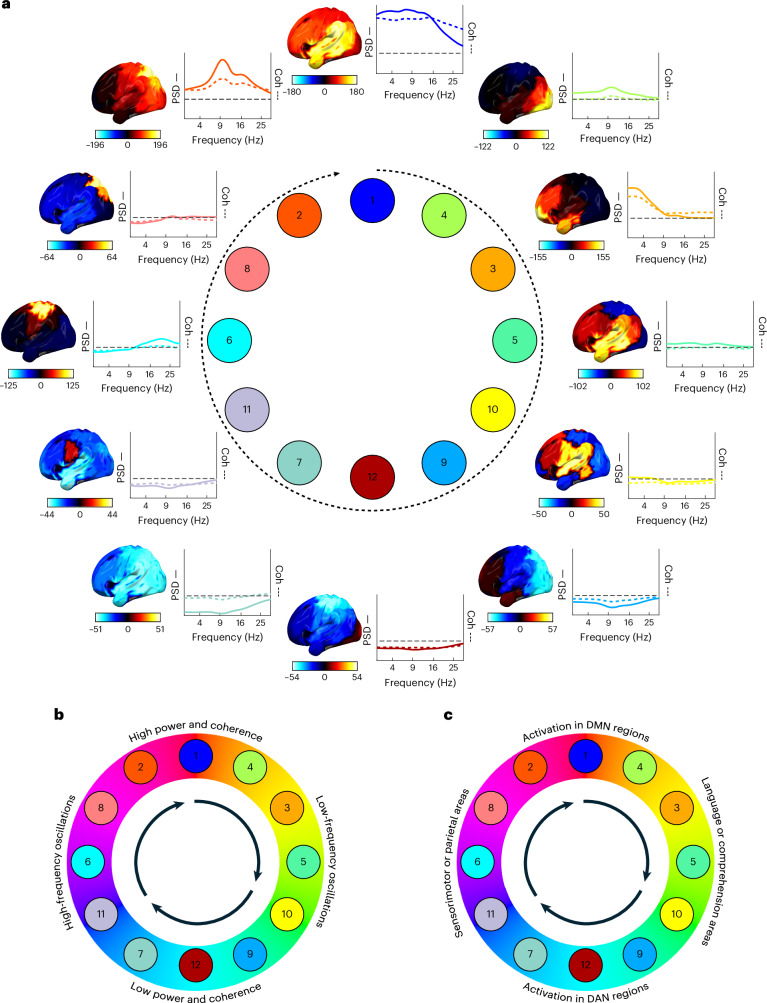
The cyclical structure groups together network states that have similar spectral properties and cognitive function in the MEG UK dataset. **a**, The spatiospectral characteristics of functional brain networks embedded in their cyclical progression. Each brain map shows the percentage increase in power (for visualization purposes, this is shown relative to the mean over states), projected on to the left hemisphere (see Supplementary Fig. 7 for the coherence maps and replication in Cam-CAN and HCP and Supplementary Fig. 1 for detailed spectral characteristics for each network state). To the right of each brain map is the spatial average PSD (solid line) and coherence (Coh; colored dashed line) as a function of frequency, relative to the average over states (horizontal dashed black line). **b**,**c**, Qualitative summary of the spectral (**b**) and spatial (**c**) modes seen in **a** (Supplementary Information Section XII). Source data

Having established that resting state networks tend to activate in a cyclical progression, we next characterized what a complete traversal of a single cycle might look like. We did this by mapping the spatial or spectral network state descriptions provided by the HMM on to the cycle. The result of this is shown in Fig. 4 for the MEG UK dataset with power maps and Supplementary Fig. 7 for the other datasets and coherence maps. We emphasize that each network state comprises a spatially defined pattern of power and coherence. To display these high dimensional representations more succinctly, Fig. 4a shows only the single dominant spatiospectral mode in each state (Methods and Supplementary Fig. 1); this information is further condensed and summarized in Fig. 4b,c. Quantitative comparisons of the MEG HMM states and the Yeo7 atlas^31^ have been made in Supplementary Information Section XII.

The first major mode of differentiation between network states emerges on the north–south axis of the clock face. States in the upper quadrant have a higher overall power and interarea coherence (that is, phase locking). States 1 and 3, in particular, show strong overlap with areas overlapping the DMN (including bilateral inferior parietal lobe, medial prefrontal cortex and medial temporal lobe; Supplementary Information Section XII). This is not a mere broadband power increase, but rather reflects different combinations of oscillatory activity in distinct or overlapping frequency bands^29^. On the lower quadrant, states have lower overall power and interarea coherence, particularly in sensorimotor and parietal areas. These states are associated with sensorimotor processing (states 9 and 12) and the DAN (Supplementary Information Section XII).

A second mode of differentiation emerges on the east-west axis of the clock face. In terms of spectral activity, network states on the left of the quadrant display activity in higher-frequency bands, for instance, state 6 is associated with β-band (14–30 Hz) activity and state 2 with α-band (7–13 Hz) activity. On the other hand, states on the right-hand side show activity in lower-frequency bands, particularly the δ (1–4 Hz) and θ (4–7 Hz) bands. Spatially, states on the left-hand side show increased low-frequency activity in sensorimotor and parietal areas, which are associated with sensorimotor inhibition. Meanwhile, states on the right-hand side show activity mostly in frontotemporal and language areas^2,29,32^.

The differentiation in spatiospectral activity suggests that different types of brain function and processes are localized to particular phases of the cycle, for example, these results suggest that network states going into the DMN are linked to sensorimotor inhibition through increased α or β power. In contrast, networks going away from the DMN comprise slower-frequency content in higher-order frontotemporal areas, which is followed in turn by low-power sensorimotor states, in particular state 7, characterized by a decrease in oscillatory power in the parietal regions overlapping the DAN.

In the interest of reproducibility, this plot has been replicated on the Cam-CAN and HCP datasets. The main findings summarized in Fig. 4b,c have been reliably reproduced (Supplementary Fig. 7), despite some moderate differences in network state definitions.

### Cycle statistics relate to cognition and demographics

**Fig. 5:**
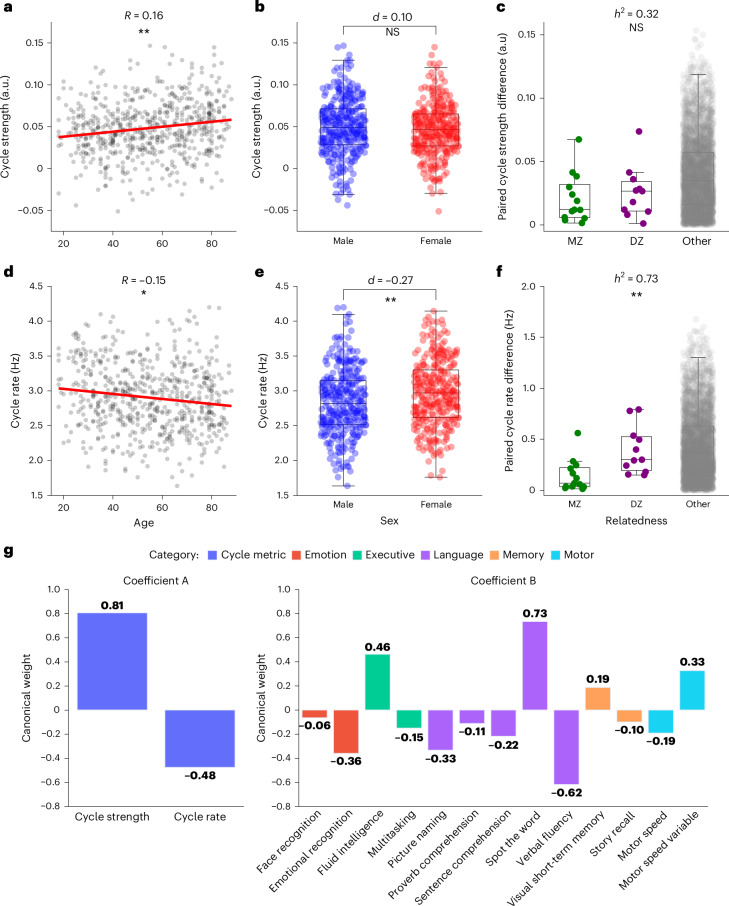
Cyclical activation statistics relate to individual traits and are heritable. **a**, Cycle strength predicting age in Cam-CAN (*n* = 609; Student’s *t*-test, two sided, *β* = 2.49, s.e. = 0.75, *t*(605) = 3.30, *P* = 0.0010; post hoc Pearson’s correlation *R* = 0.16). **b**, Cycle strength not differing in males and females (*n* = 609; Student’s *t*-test, two sided, *β* = −0.052, s.e. = 0.085, *t*(605) = −0.61, *P* = 0.54; Cohen’s *d* = −0.10). **c**, The absolute difference in cycle strength across MZ twins, DZ twins or unrelated pairs in the HCP (*n* = 79). The cycle strength is not significantly heritable (*n* = 79; ACE test, *h*^2^ = 0.32, 95% CI = 0.01–0.67, *P* = 0.12). **d**, Cycle rate predictive of age (*n* = 609; Student’s *t*-test, two sided, *β* = −2.04, s.e. = 0.75, *t*(605) = −2.71, *P* = 0.0070; post hoc Pearson’s correlation *R* = −0.15). **e**, Cycle rate differing in males and females (*n* = 609; Student’s *t*-test, two sided, *β* = 0.24, s.e. = 0.086, *t*(605) = 2.81, *P* = 0.0050; Cohen’s *d* = 0.27). **f**, Heritable cycle rate (*n* = 79, ACE test, *h*^2^ = 0.73, 95% CI = 0.29–0.98, *P* = 0.0039). The circles correspond to individual participants (**a**, **b**, **d** or **e**) or pairs of participants (**c** and **f**), the boxplots display the median and 25th and 75th percentiles and the whiskers indicate the minimal and maximal values not considered as outliers. ^*^P < 0.05, ^**^P < 0.01, ^***^P < 0.001. **g**, Canonical weights from the second (significant) pair of canonical variates. Left: cycle metrics; right: cognitive scores. See Supplementary Information Section IX for evidence that these results are robust to minor changes in metric definitions and Supplementary Information Section XIII for the other canonical variate variables and post hoc Pearson’s correlations between individual variables. Source data

Inspired by this qualitative segmentation of cycles into four ‘metastates’ of distinct spatiospectral characteristics, we defined a full-cycle traversal as the sequential activation of these (see Methods and Supplementary Information Section VIII for details). This allowed us to define cycle duration as a metric to summarize the timescale of these dynamics. Cycle duration was, on average, on the timescale of 300–1,000 ms (MEG UK mean (*μ*) (s.d.) = 549 (154) ms; Supplementary Information Section VIII: Cam-CAN *μ* (s.d.) = 355 (62.4) ms; Fig. 5d: HCP *μ* (s.d.) = 528 (104) ms). We could then relate cycle duration, or in fact its more normally distributed inverse (that is, cycle rate), to individual traits, together with the previously defined cycle strength.

We first made sure that the cycle strength and cycle rate are consistent within individuals. We computed the intraclass correlation coefficient (*R*) on the metrics for the three sessions per participant available in the HCP dataset. This confirmed that both metrics are consistent across sessions (cycle strength: *R* = 0.43 (95% confidence interval (CI): 0.29–0.56), *F*-statistic *F*(78,158) = 3.2, *P* = 1.9 × 10^−10^; cycle rate: *R* = 0.80 (95% CI: 0.72–0.86), *F*(78,158) = 12.9, *P* = 0). We also found that these metrics are robust to the number of network states fitted in the first-level HMM (Supplementary Information Section IX). We then took advantage of the large and equally distributed age range (18–86 years) and sex in the Cam-CAN dataset and asked whether either could be predicted by cycle strength or cycle rate (Fig. 5). As both age and sex are known to affect heart rate, and the heartbeat has a strong effect on the MEG signal, we first regressed out heart rate. Next, we fitted a generalized linear model, which revealed that cycle strength reliably predicted age (*β* = 2.49, s.e. = 0.75, *t*(605) = 3.30, *P* = 0.0010, post hoc Pearson’s correlation *R* = 0.16), but not sex (*β* = −0.052, s.e. = 0.085, *t*(605) = −0.61, *P* = 0.54, Cohen’s *d* = −0.10), and cycle rate predicted age (*β* = −2.04, s.e. = 0.75, *t*(605) = −2.71, *P* = 0.0070; post hoc Pearson’s correlation *R* = −0.15) and sex (*β* = 0.24, s.e. = 0.086, *t*(605) = 2.81, *P* = 0.0050, Cohen’s *d* = 0.27). These findings are robust to minor changes in the way that metrics are defined (Supplementary Information Section IX). We replicated these correlations in the other datasets and confirmed the correlations between age and cycle rate and strength but found no statistical difference between males and females (Supplementary Information Section X). A post hoc analysis revealed that the correlation between age and cycle strength can be explained by a combination of (1) stronger pairwise asymmetries between network states on average and (2) fewer deviations from the cycle structure (that is, fewer backward or random transitions) (Supplementary Fig. 11).

The correlations between cycle metrics and age suggest that older people have slower and stronger cycle dynamics. Given that cognitive slowing and inflexibility are often observed in older people^14,33^, we wondered whether these were related. We first regressed out age, sex and heart rate from all variables and then used a canonical correlation analysis (CCA) to find a relationship between cycle metrics and cognitive scores, resulting in two orthogonal, canonical correlation variates. This confirmed a statistically significant relationship between cognitive scores and cycle metrics for the second (*R* = 0.17, *F*(12, 597) = 1.51, *P* = 0.0087 versus permutations; Fig. 5g), but not the first (*R* = 0.19, *F*(26, 1,192) = 1.54, *P* = 0.19; Supplementary Fig. 15) variate. Notably, the canonical weights of cycle metrics for the significant relationship with cognitive scores were in the opposite direction and so was the correlation between these metrics and age. This could suggest a relationship between cycle dynamics and age-related cognitive decline. Replication of this finding was not assessed in other datasets because comparable cognitive scores were not available.

We next wondered whether cycle metrics could be genetically determined. The Cam-CAN dataset did not allow us to test this for lack of twin data, so we turned to the HCP dataset, which contains data of monozygotic (MZ) and dizygotic (DZ) twins and unrelated pairs of participants. using an ACE model of heritability^34,35^. The ACE aims to partition the phenotypic variance into three components: additive genetic variance (A), shared environmental factors (C) and nonshared environmental factors (E). Despite the relatively small cohort of twin data, we found strong evidence that cycle rate, but not cycle strength, is heritable (Fig. 5c,f). In fact, 73% of the variance in cycle rate in the population could be explained by genetic factors (*h*^2^ = 0.73, 95% CI = 0.29–0.98, *P* = 0.0039). We did not find such an effect for cycle strength (*h*^2^ = 0.32, 95% CI = 0.01–0.67, *P* = 0.12) nor did we find evidence that environmental factors affected cycle metrics (cycle strength: the amount of variance explained by environmental factors (*c*^2^) = 0.18, 95% CI = 0–0.41; cycle rate: *c*^2^ = 0, 95% CI = 0–0.43). To make sure that demographic or morphometrics factors did not bias these results, we systematically regressed out potential confounds (for example, age, sex, brain volume; Methods and Supplementary Fig. 10). The heritability estimate of cycle rate remained high (*h*^2^ = 0.68) and significant even with the most stringent confound modeling.

### Cycles are preserved in task data and behaviorally relevant

Having established that cortical networks activate in cycles across multiple datasets in a manner predictive of individual traits, it remained possible that they nevertheless reflect some neurophysiological feature of little or no relevance to cognitive processes. We therefore first asked whether the cyclical patterns observed during rest were related to spontaneous memory replay. Second, we tested whether cyclical patterns persisted in task data and whether variance in cyclical metrics over task epochs related to variance in task performance.

**Fig. 6:**
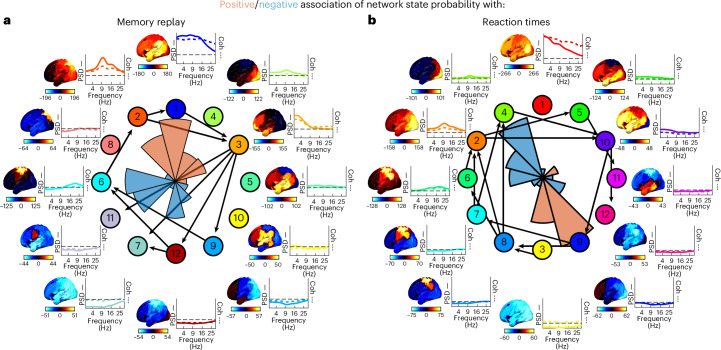
Cycle phase is predictive of cognitive function. **a**,**b**, Cycle dynamics in a memory replay dataset^24,36^ (**a**) and a visuomotor task dataset^37^ (**b**). Positive or negative associations of the network state probability with memory replay (**a**; ranging from 0% to 7% increased (red) or decreased (blue) probability) and reaction times (**b**; correlations ranging from −0.11 (blue) to +0.11 (red)) are indicated by polar histogram insets. The colored solid lines indicate the PSD and the colored dashed lines the coherence. Source data

In the memory replay^36^ dataset, participants learned sequence structures between different visual images. The representations of these have been shown to replay spontaneously during a subsequent rest period^36^ and recent work has shown that states 1–4 in particular co-activated with memory replay^24^, whereas most other network states were less likely to be active. Here we found cycle structure to persist in this dataset (Fig. 6a; cycle strength, mean (s.d.): *S* = 0.017 (0.017), *P* < 0.001, versus permutations) and, interestingly, that those network states that have previously been shown to be positively correlated with memory replay are clustered in the north face of the circle, whereas the strongest negatively correlated states are on the opposite phase (Fig. 6a, polar histogram).

These results suggest that these internally generated memory replay events might be precisely timed with respect to the phase of the cyclical activity. However, memory replay does not involve any exogenously prompted behavior. In particular, it is possible that external events or active behavior dictate network state activations such that cyclical activity disappears. To answer this question, we applied TINDA to a visual task dataset^37^.

In the Wakeman–Henson faces dataset^37^, 19 participants saw a series of famous, unfamiliar or scrambled faces in 6 sessions and had to report their asymmetry with a button press. This dataset has previously been shown to elicit task-dependent network dynamics^38^. Despite the trial structure, we again confirmed the presence of an overall cyclical structure in network state activation (*S* = 0.058 (0.031), *P* < 0.001 versus permutations) and we also observed that the ordering of states along the cycle replicated that of the MEG UK dataset (Δ*θ* = 0.84 rad, *P* = 0.027; Fig. 6b). We then correlated the state time courses time locked to button press with the reaction times for each trial. Network probability at 500 ms before button onset in each individual was significantly positively correlated with their reaction times in state 3 (*R* = 0.069, 95% CI = 0.031–0.11, *t*(18) = 3.8, *P* = 0.0014; Student’s *t*-test against zero) and state 9 (*R* = 0.11, 95% CI = 0.051–0.17, *t*(18) = 3.9, *P* = 0.0009) and negatively correlated with state 2 (*R* = −0.058, 95% CI = −0.034 to −0.083, *t*(18) = −5.0, *P* = 0.0001), state 4 (*R* = −0.094, 95% CI = −0.056 to −0.13, *t*(18) = −5.2, *P* = 0.0001) and state 6 (*R* = −0.051, 95% CI = −0.023 to −0.078, *t*(18) = −3.9, *P* = 0.001). Notably, as in the Replay dataset, the positive and negative correlations, respectively, clustered on opposite sides of the cycle (Fig. 6b, polar histogram). In particular, if there were a high probability that a low-power state was active 500 ms before the button press, responses would be slower, and vice versa for high-power visual or attentional states. Furthermore, when we estimated cycle strength on a trial-by-trial basis (that is, by running TINDA on the 3-s segment before a button press), we found a small, but significant Pearson’s correlation between the cycle strength and reaction times over trials (*R* = −0.025, 95% CI = −0.011 to −0.040, *t*(18) = −3.8, *P* = 0.0014), such that higher cycle strength was associated with faster responses. Together, these results indicate that cycle dynamics on a moment-to-moment basis are relevant for cognition.

## Discussion

### Summary

We showed that the activations of a canonical set of large-scale cortical networks are organized in an inherently cyclical manner, where networks are activated at a preferred phase in a periodic cycle. Furthermore, we showed that the cycle’s period and integrity relate to age and cognition, whereas cycle phase is predictive of behavior on a moment-to-moment basis. Together, these results suggested that cyclical activation of functional brain networks might ensure a periodic activation of essential cognitive functions.

### Organizational structures in functional brain networks

Previous research in fMRI has shown a dissociation of RSNs into cognitive and perceptual clusters or ‘metastates’^14,39–41^. In particular, states within the perceptual or cognitive clusters were highly correlated in terms of temporal occurrence^14,39^ and connectivity profile^41^, but not in the states between clusters. Although fMRI and MEG have different biophysical origins and temporal sensitivity, the spatial extent of RSNs is remarkably similar^11,17,38^. Our results indeed suggest a dissociation of perceptual and cognitive network states, by positioning them on opposite phases of the cycle, most clearly observed for the DMN and DAN (Fig. 3). Moreover, it suggests a preferred pathway of state transitions between these extrema.

### Broken detailed balance in brain activity

Network state transition asymmetries like these have further been linked to macroscopic broken detailed balance. This deviation from thermodynamic equilibrium is a hallmark of living systems and can be directly linked to energy consumption and system complexity^42^. Previous research in this field has shown that the level of broken detailed balance correlates with the level of consciousness^20,21^ and cognitive exertion^19,22^, and has potential as a biomarker for progressive brain disorders^43^. Here we add insights into the fast transient networks of oscillatory power and synchronization underlying macroscale, broken detailed balance and revealed the timescale at which these cognitively relevant networks cycle. Furthermore, we found that macroscale, broken detailed balance increased with age and was stronger on longer timescales, although it is unknown how different methodological approaches interrelate and provide insights into the temporal sensitivity of broken detailed balance.

### Motifs in brain networks

Although prior research has established that transitions between brain network states are not random, identifying phenomena like ‘asymmetric transitions’, ‘repeated motifs’ or potentially localized ‘cyclical motifs’^44,45^, these findings differ fundamentally from the ‘cyclical pattern’ investigated in this paper. Previous work typically highlighted specific, often localized, aspects of network dynamics: asymmetrical transitions show a preferred direction between two states (A → B is more likely than B → A) and repeated or cyclical motifs might reveal recurring short sequences or small loops involving a subset of networks. For example, Sporns and Kötter showed that some motifs are more prevalent in anatomical connectomes than in random networks, some of which are cyclical^44^. However, none of these necessarily imply the existence of a global, overarching cycle that incorporates a full set of canonical networks in a specific, repeatable order. For example, strong asymmetry between a few states or the existence of a small recurring motif, like A → B → C → A (for example, VP → V3 → V2 → VP in the macaque visual cortex^46^), does not guarantee that the system tends to progress through all other major network states, for example, D, E, F, in a consistent sequence before returning. The current study’s cyclical pattern posits this more comprehensive, large-scale temporal organization, suggesting that the brain tends to flow through the full set of recognizable, canonical, large-scale cortical networks over hundreds of milliseconds, a distinct concept from previously described local transition biases or mini-sequences.

### Timescales of structured brain dynamics

Previous studies investigating the asymmetry in functional brain networks have focused on either Markovian state transitions^11,22^ or the (time-lagged) correlation between network activation patterns^2,5,14,19^. TINDA differs from these by considering the general pattern in network transitions beyond the direct (that is, Markovian) transitions. This revealed that asymmetrical network transitions occur to a different extent at different timescales, with strongest asymmetries on >2 s timescales (Fig. 3). These asymmetries described an overall cyclical activation pattern, which, due to the stochasticity of individual cycles, had lower typical durations of 300–1,000 ms (Fig. 5 and Supplementary Information Section VIII), an order of magnitude larger than the typical lifetime of a cortical network state^11^. These timescales have previously been shown to be a lower limit for scale-free global brain dynamics^1^ and the most relevant timescale for global brain processing^47,48^. Although we have shown that cycle dynamics at these temporal scales are relevant for behavior on a moment-to-moment basis (see below), future work should further explore their role in different cognitive tasks and at different temporal scales.

### Cycle dynamics and age

It is interesting that we found these timescales to lengthen with age, concurrent with an increase in cycle strength. This is in line with age-related cognitive decline and slowing^49^, although correlations with cognitive performance indicated a more complex relationship. Other age-dependent changes in brain activity are ubiquitous and include a slowing in the power spectrum^50–52^ and a decrease in network connectivity, which has been related to a decrease in the segregation of functional networks^53–57^.

### Heritability of cycle metrics

Another observation that argues for cyclical dynamics to be rooted in our biology is its strong genetic component of cycle rate. Other heritable components to large-scale brain networks have been shown in the past, including connectivity in specific functional networks^14,58–61^, frequency bands^62^ and static connectivity^63^. In particular, Vidaurre et al. found that the degree to which an individual spent more time in either perceptual or cognitive fMRI resting states was heritable^14^. How these and other fMRI dynamics are related to the cyclical dynamics described here is a topic for future research.

### Relevance for cognition

Although most datasets that we explored here involved wakeful rest, the cyclical dynamics also persisted in task data. Moreover, the phase within the cycle and cycle strength were predictive of cognitive function. Although the HMM framework has successfully shown large-scale cortical network associations with cognitive function before^24,38^, here we add that positive and negative associations with memory replay, or response speed, were predicted by network states on oppositive phases of the cycle. One question that arises is whether cycle dynamics like speed and phase can be (consciously) controlled or disrupted by a cognitive task, which is expected from the stochasticity of state transitions within the cycle. On the other hand, the persistence of the ordering of network states within the cycle and the detrimental effect of cycle phase on certain cognitive functions suggests that it could reflect a homeostatic process. In fact, homeostatic cyclical rhythms are omnipresent in biological systems^64^, with the sleep cycle being one of the most well-known examples^65^. In sleep, cycling through each of the five functional stages allows the body to experience the benefits of each stage multiple times throughout the night, ensuring that each function is carried out even if sleep is disrupted. Similarly, cycles in large-scale brain networks could ensure periodic activation of essential cognitive functions, with stochasticity enabling cognitive flexibility.

### Limitations and future directions

The present study comes with a number of limitations. First, the TINDA method is a post hoc analysis tool that is used on binarized state time courses (that is, brain networks are either ‘on’ or ‘off’) and, furthermore, it does not incorporate an explicit model of long-term (variable time) state transitions. In future work, we hope to deploy non-Markovian models like neural networks for inferring brain networks (such as DyNeMo^66^), but it remains an open question how to adapt TINDA to state time courses that are not mutually exclusive. Although we have reproduced our main results in multiple datasets, some results could not be reproduced, that is, the heritability of cycle metrics and the association of cycle metrics with cognitive scores. Replication of these analyses in independent datasets is essential but relies on the availability of the relevant data. This would also clarify the role of cycle dynamics for cognition across individuals and its potential as a biomarker for disease. Another limitation of the current study, and the field of functional brain networks more generally, is a lack of taxonomy with respect to the macroscale functional brain networks. This can lead to ambiguity or overinterpretation of the functional network and it is unclear in what capacity they are rooted in the underlying physiology^67^. Moreover, there is no consensus in electrophysiology about which features constitute a brain network, be it coherence, power, spectral shape or how to relate these to brain networks observed in fMRI. With regard to the first point, we argue that a principled definition of a brain network is one where networks can be distinguished, not by a single arbitrarily chosen feature, but instead by multiple network features. We therefore use the time-delay-embedded (TDE) HMM^29^, in which brain networks are characterized by distinct auto-spectral and cross-spectral properties as part of a generative model that is capable of explaining the full signal content. Previous work has also shown that TDE HMM results in identifiable networks that are highly reproducible across different data, sites and task or rest designs, which validates this approach. Second, there is a growing effort to compare functional brain networks across studies^29,68,69^ and modalities^70–76^. We have made quantitative comparisons of MEG state topographies with the widely used fMRI-based Yeo7 parcellation^31^ (Supplementary Information Section XII), from which we tentatively concluded that the cycle separates the DMN (top of the cycle) and the DAN (bottom of the cycle). However, we do note that the existence and presence of cycles shown here in five independent datasets do not rely on the physiological interpretation of individual networks. More efforts need to be made in quantitatively comparing functional brain networks inferred from electrophysiology and hemodynamic responses, particularly in simultaneous recordings.

## Methods

All analyses were carried out in MATLAB^77^ and Python, using in-house-developed software packages OHBA Software Library^78–80^, HMM-MAR^81^, OSL-dynamics^69^ and MNE-Python^82,83^. The TINDA package is available for MATLAB^84^ and Python^69^.

### Data

We used data from five MEG datasets: Nottingham MEG UK (*n* = 55), Cam-CAN (*n* = 612), HCP (*n* = 79), Replay (*n* = 43) and Wakeman–Henson (*n* = 19). MEG UK, Cam-CAN, HCP and Replay contain MEG resting state data, Replay and Wakeman–Henson MEG task data and all but the Replay dataset include T1-weighted MRI brain scans. Datasets include demographic data and, in the case of HCP, heritability data. Ethics and consent details are outlined separately for each of these datasets below. No additional ethical approval was required for the present study.

### MEG UK

The UK MEG partnership data comprised 77 healthy participants recruited at the University of Nottingham, of whom 55 were used after discarding 22 because of excessive head movements or artifacts. The dataset contains structural MRI scans and MEG data from a CTF MEG system containing 275 axial gradiometers with a sampling frequency of 1,200 Hz. The participant group used in this analysis had a mean age of 37.8 years (range 19–62 years), 29 of whom were female and 26 male. All participants gave written informed consent and ethical approval was granted by the University of Nottingham Medical School Research Ethics Committee. The MEG data comprised roughly 5–6 min of eyes-open resting state and have previously been used to characterize MEG RSN dynamics^24,29,85^.

### Cam-CAN

The Cam-CAN dataset comprised data of 700 healthy participants recruited at the University of Cambridge, of whom 612 were used here. The dataset contained structural MRI scans and MEG data from an Elekta Neuromag Vectorview system containing 102 magnetometers and 204 orthogonal planar gradiometers, with a sampling rate of 1 kHz. The participants’ mean age was 54.6 (range 18–88) years, with 83–95 participants per age decile (except in the 18th to 28th decile, which counts for 45); 310 were male and 302 female, equally distributed across the age deciles. All participants gave written informed consent and ethical approval was granted by the Cambridgeshire Research Ethics Committee. The MEG data comprised approximately 9 min of eyes-closed resting state.

### HCP

The MEG component of the HCP comprised 100 healthy participants recruited at the Saint Louis University, of whom 79 were used after discarding participants with excessive variance. The dataset contains structural MRI scans and MEG data from a 4D Neuroimaging MAGNES 3600 MEG system containing 248 magnetometers sampled at 2 kHz. The participant group had a mean age of 29 (range 22–35) years, of whom 37 were females and 42 males and contained data of 13 MZ twin pairs and 11 DZ twin pairs. All participants gave written informed consent and ethical approval was granted by the local ethics committee. The MEG data comprised 3× 6 min of eyes-open resting state.

### Replay

The Replay data^36^ contained a primary dataset (dataset 1) and a replication dataset (dataset 2). For both datasets, participants were scanned on a 275-channel CTF MEG system while engaged in a localizer task, a sequence learning task and periods of rest. Activations corresponding to images in the localizer task were found to replay during rest, in the sequence that corresponded to the learned sequences. The top 1% replay probabilities were here defined as the memory replay events, as in Higgins et al.^24^. Replay dataset 1 was acquired from 25 participants with a mean age of 24.9 (range 19–34) years, of whom 11 were males and 14 females. Four participants were excluded due to large motion artifacts or missing trigger information. All participants signed written consent in advance. Ethical approval for the experiment was obtained from the Research Ethics Committee at University College London under ethics no. 9929/002. Replay dataset 2 was acquired from 26 participants with a mean age of 25.5 (range 19–34) years, of whom 10 were males and 16 females. Four participants were later excluded due to motion artifacts or failure to complete the task. All participants signed written consent in advance. Ethical approval for the experiment was obtained from the Research Ethics Committee at University College London under ethics no. 9929/002. In the present study, Replay datasets 1 and 2 were analyzed jointly.

### Wakeman–Henson dataset

The Wakeman–Henson faces dataset^37^ comprised MEG data acquired on an Elekta Neuromag Vectorview system of 19 participants. Of these, 8 were female and 11 male and the age range was 23–37 years. All participants gave written informed consent and ethical approval was obtained from the Cambridge University Psychological Ethics Committee. Each participant completed six sessions of a perceptual task in which they would see a famous, familiar or scrambled face, to which they had to respond based on the symmetry of the image. Each trial began with a fixation cross onset between 400 ms and 600 ms before a target stimulus appeared. The target was either the face or scrambled face stimulus and remained on-screen for between 800 ms and 1,000 ms. Further details can be found in ref. ^37^.

### Preprocessing

MEG data were co-registered to the MRI structural scans or to fiducial markers in the Replay data where MRI structural scans were not available. The MEG UK and Cam-CAN data were downsampled to 250 Hz, filtered in the 1-Hz to 45-Hz range (using zero-phase digital filtering so that effects were symmetrical across time) and source reconstructed using an LCMV beamformer to 3,559 dipoles. The dipoles were then combined into 38 parcels spanning the entire cortex by taking the first principal component of all dipoles in a parcel. This parcellation was used previously to estimate large-scale static functional connectivity networks in MEG^50^. The HCP data were downsampled to 240 Hz, filtered in the 1-Hz to 80-Hz range and source reconstructed using an LCMV beamformer to 5,798 dipoles. The dipoles were then combined into 78 parcels of the automated anatomical labeling parcellation^86^, spanning the entire cortex by taking the first principal component of all dipoles in a parcel. Bad segments were removed manually and correction for spatial leakage was applied using symmetrical multivariate leakage correction^87^. Finally, the potential inconsistency over participants of ambiguous source polarity was removed using sign flipping based on lagged partial correlations^38^.

### Hidden Markov modeling

To find large-scale brain networks in a data-driven way, we applied a TDE HMM with 12 states and 15 embeddings, corresponding to lags of −28 ms to +28 ms (−29 ms to +29 ms for HCP). Note that we referred to the HMM states as ‘network states’ to reflect the method being designed and that it has been shown to find states that represent distinct cortical networks of oscillatory brain activity in MEG or electroencephalographic data^29^. The HMM framework is a generative model that assumes that there are a finite number (*K*) of recurring, transient and mutually exclusive hidden states that generate the observed data. Here each state is characterized by a spatiospectral profile (that is, in terms of power spectral density (PSD) and connectivity in or across regions). Thus, every time point in the data was associated with one of the states [1, 2, … *K*], and the sequence of states was assumed to be Markovian. This meant that the state active at time point *t* depended only on the state active at *t* − 1, captured by the transition probability between all states. We used a multivariate Gaussian observation model with zero mean. Models were inferred separately for the MEG UK, Cam-CAN and HCP datasets. For the Replay datasets, we kept the model from the MEG UK dataset fixed and subsequently fitted it to the Replay data, as in Higgins et al.^24^.

### Spectral analysis

We estimated the spectral information (PSD and coherence) for each state by fitting a multitaper to the original, parcellated data, condition on the active functional brain network, as in Vidaurre et al.^29^. The multitaper used a taper window length of 2 s and a frequency range of 1–45 Hz with a 0.5-Hz resolution (that is, applying 7 Slepian tapers). This reflected the full multivariate model parameter space (an array that was frequencies × channels × channels × states); however the high model dimensionality necessitated further dimensionality reduction methods if this information were to be visualized. We reduced the spectral dimensionality using spectral mode decomposition, resulting in spatial power and coherence maps for a data-driven set of frequency band modes. This decomposition was implemented by non-negative matrix factorization^29,38^. We fitted this with two modes to separate wideband activity from high-frequency noise (Supplementary Fig. 1). We then used the wideband mode to weight the frequencies of the individual states when producing topographies.

### Ordering the HMM states

We ordered the HMM states based on state coherence using the MEG UK dataset. States inferred from the MEG UK dataset were reordered based on the mean coherence in that state, from high (state 1) to low (state 12) coherence. The ordering in the other datasets (Cam-CAN, HCP and Wakeman–Henson) was then matched to the MEG UK ordering as follows. First, the correlation was computed between the coherence of each pair of states, where a ‘pair of states’ consisted of one state from MEG UK and one from, for example, Cam-CAN. The correlations were then used as a cost function to solve the linear assignment problem using the matchpairs function in MATLAB^88^, matching every state in, for example, the Cam-CAN dataset to a state in the MEG UK dataset. Due to the different parcellation used in the HCP dataset, here we used the correlation between power maps in MNI volume space as the cost function. In figures throughout the manuscript, state numbers thus indicate equivalent (that is, ‘best matching’) states, whereas state colors were different between datasets, to stress that state descriptions were inferred independently for each dataset.

### TINDA

We developed the TINDA method to analyze interstate dynamics in the context of dispersive ISIs. We first partitioned all observed ISIs, defining ${{T}_{1}}^{m,i}$ to be the set of timepoints that fall within the first half of ISIs for state *m* and ${{T}_{2}}^{m,i}$ to be the set of all timepoints that fall within the second half of these ISIs. We then computed the *K* × *K* fractional occupancy (FO) asymmetry matrix (*A*), defined as the matrix with (*m*,*n*)th entry as:

$${A}_{m,n}={ < {{{\mathrm{FO}}}}_{{T}_{1}^{\;n}}-{{{\mathrm{FO}}}}_{{T}_{2}^{\;n}} > }_{m}$$

where < … >_*m*_ denotes the average FO difference for state *n* over state *m* ISI’s (Fig. 1). These FO asymmetry matrices are computed for each participant.

### Cycle detection and cycle strength

TINDA establishes whether there is a general flow of states into and out of a particular state. We investigated whether this pattern is embedded in a larger, hierarchical structure, specifically a cycle. We interpret the FO asymmetry matrix, *A*, as a weighted, directed graph of *K* nodes (that is, number of states) and *K*^2^ − *K* edges (that is, from every node to every other node). The FO asymmetry thus defines the weight and direction of each edge.

To investigate how these edges relate to specifically cyclical dynamics, we defined a metric of cycle strength for each configuration of the *K* nodes around the unit circle. Each node is associated with a phase *q*, positioned on the unit circle in 2*p*/*K* intervals, spanning [0, 2*p*]. We could then represent each directed transition, from state *n* to state *m*, by a vector in the complex plane defined by the phase difference between the relative position of nodes *m* and *n*:

1 $${d}_{m,n}={e}^{i({q}^{m}-{q}^{n})}$$

The magnitude of the imaginary component of this vector represents a geometric projection of each state transition onto the plane tangential to the unit circle at node *n*. Trivially, the cumulative sum of these vectors for all *n* and *m* states is zero. However, if these vectors are weighted by the strength (and direction) of the corresponding FO asymmetry, then the sum of their imaginary components represents the cumulative strength (that is, of the asymmetry) and the polarity represents the net direction (that is, clockwise (+) versus counterclockwise (−)) of transitions tangential to the unit circle. Hence, we define the cycle strength, *S*, as:

2 $$S=-\beta \times \sum _{m}\sum _{n\ne m}{A}^{m,n}\times \sin \left({q}^{m}-{q}^{n}\right)$$

where *β* is a normalization factor based on the theoretical maximum cycle strength for *K* states, such that *S* is constrained to be [−1, 1]. The theoretical maximum cycle strength is computed for *K* states by assuming a perfect asymmetry of +1 for all possible clockwise connections and −1 for all possible negative connections.

We permuted the position of each state on the unit circle (that is, the node identity) to maximize *S*. This revealed the sequence of states for which the overall directionality is maximal in the clockwise direction. Note that we could have chosen to maximize negative *S* instead. This would have resulted only in all circle plots going in a counterclockwise direction; it would not have changed any of our results.

### Circle plots

The circle plots all show the network states in the sequence that maximizes the cycle strength (in a clockwise direction). The edges *E* that are shown are those where the FO asymmetry is statistically significant (ʽStatisticsʼ) and the direction of the arrow depends on the sign of the corresponding edge asymmetry, where *α* is the (corrected) statistical threshold (ʽStatisticsʼ):

3 $${E}^{j,k}=\left\{\begin{array}{l}\;\;\;0,\,\qquad{p}_{j,k}\ge \alpha \,\\-1,\qquad{p}_{j,k} < \alpha \wedge {A}^{j,k} < 0\\ +1,\,\quad\;\;{p}_{j,k} < \alpha \wedge {A}^{j,k} > 0\end{array}\right.$$

### Cycle rate

To quantify cycle rate, we applied a post hoc analysis to the state time course parameters already learned. Specifically, we derived a feature from the state time courses defined as the number of state visits in a sliding window equal to the average state lifetime (64–68 ms, depending on the dataset). We then fitted a second-level HMM to this feature time course, where this HMM used Poisson’s observation model and sequential Markov dynamics^89^. We selected a model with *K* = 4 states, where we initialized the state probabilities as the distance (that is, in circle space) to the centroid of each of the four modes in Fig. 4b,c. We also enforced a sequence of state 1 > state 2 > state 3 > state 4 > state 1, and so on, such that a single cycle was defined as sequential activation of each of the four modes in Fig. 4b,c. Using this initialized model, we inferred the state time courses from the data without training the model to convergence. This was done to not deviate from our definition of a ‘cycle’ and subsequently to quantify cycle duration as the time that it takes to cycle through a full 1 > 2 > 3 > 4 sequence of the second-level HMM. For correlations with individual traits, the inverse (that is, cycle rate) was used, because this was more normally distributed.

### Statistics

#### FO asymmetry and circle plots

Circle plots show the edges where the FO asymmetry is strongest (and significant). To test for significance, the FO asymmetry was tested on the group level with a two-tailed, dependent sample Student’s *t*-test for each of the connections *m* and *n*, where the *α* threshold of 0.05 was Bonferroni corrected for 132 tests (that is, *K*^2^ − *K*), resulting in a corrected *α* threshold of 0.00038. Due to the large numbers of participants in the HCP and Cam-CAN, more stringent thresholds were applied in these datasets. For the Cam-CAN, the edges with absolute *t*-values >11 are shown (corresponding to *P* < 3.8 × 10^−24^) and, for the HCP dataset, the edges with absolute *t*-values >4.3 (corresponding to *P* < 3.8 × 10^−5^).

#### Cycle strength

We reported the results for the sequence of network states that results in the strongest, clockwise cycles. A claim of nonzero cycle strength could be a trivial consequence of this optimization. For this reason, we compared the observed cycle strength with that from permutations, where, for each permutation, we permuted each participant’s state labels and recomputed the FO asymmetry and optimized state ordering. This was done 1,000×. The observed cycle strength was compared with the permuted versions at *α* = 0.05.

#### Within-subject consistency of cycle metrics

The individual consistency of cycle metrics was directly estimated using the intraclass correlation coefficient in MATLAB, with type ‘1-1’ as implemented in Salarian^90^.

#### Correlation with individual traits

For the correlation of cycle rate and cycle strength with individual traits, we first regressed out heart rate. Outliers >3 s.d. from the mean value were removed and cycle metrics were normalized, before the generalized linear model. We fitted a mean term and the cycle rate and cycle strength to age and sex separately, using a Gaussian and binomial distribution, respectively. The *β* terms for each were significant if the corresponding *P* value was lower than the *α* threshold of 0.0125 (that is, 0.05 corrected for 4 tests).

#### Heritability

To test whether variance in cycle metric could be explained by genetic factors, we used an ACE model, as implemented in the Accelerated Permutation Inference for the ACE model (APACE) framework^91^. APACE was run on all participants’ cycle metrics for the three resting state sessions, separately for cycle rate and cycle strength, using 10,000 permutations. The *α* thresholds of 0.05 were Bonferroni corrected for 2 tests. To ensure that estimated heritability effects were not caused by common demographic and morphometric measures, we repeated the analysis after regressing out the following confounds in stepwise fashion (Supplementary Information Section IX): age, the square of age, sex, an age and sex interaction, an interaction between sex and the square of age and the cube root of intracranial volume and cortical volume (both estimated using FreeSurfer^92^).

#### Correlation with cognitive scores

A CCA was executed on the Cam-CAN dataset, between the cyclical summary metrics (cycle rate and strength), on the one hand, and 13 cognitive scores, on the other. For all metrics, we first regressed out age, sex and heart rate, and then *z*-transformed the data. The CCA resulted in two CCA components, which were tested for significance by comparing against a permutation distribution of 10,000 permutations, where, for each permutation, the cognitive scores were shuffled over participants.

#### Correlations with reaction times in the Wakeman–Henson data

We time locked the state probability time courses to the button presses in the Wakeman–Henson data and correlated state probability at 500 ms before the button press with the reaction time on that trial. This was done separately for each session and state, after which we averaged the correlations over sessions for each participant. We tested whether the correlation was significantly different from zero for each state with a paired Student’s *t*-test, using a Bonferroni-corrected *α* level of 0.05/*K*. Similarly, we correlated reaction times with an instantaneous estimate of cycle strength, computed by running TINDA on 3-s segments before the button press and calculating the cycle strength. Correlations were tested against zero on the group level using a paired Student’s *t*-test.

### Reporting summary

Further information on research design is available in the Nature Portfolio Reporting Summary linked to this article.

## Online content

Any methods, additional references, Nature Portfolio reporting summaries, source data, extended data, supplementary information, acknowledgements, peer review information; details of author contributions and competing interests; and statements of data and code availability are available at 10.1038/s41593-025-02052-8.

## Supplementary information

Supplementary Information Supplementary Information Sections I–XIII and Figs. 1–15.

Reporting Summary

Supplementary Video 1 Summary of findings.

## Source data

Source Data Fig. 1 Statistical source data.

Source Data Fig. 2 Statistical source data.

Source Data Fig. 3 Statistical source data.

Source Data Fig. 4 Statistical source data.

Source Data Fig. 5 Statistical source data.

Source Data Fig. 6 Statistical source data.

## Data Availability

The MEG UK Partnership data are held by the MEG UK Partnership, for which access can be requested at https://meguk.ac.uk/database. The Cam-CAN dataset is available upon request to https://camcan-archive.mrc-cbu.cam.ac.uk/dataaccess/datarequest.php. The HCP dataset is freely available at https://db.humanconnectome.org/app/template/Login.vm, but will require an application for sensitive data (https://www.humanconnectome.org/storage/app/media/documentation/LS2.0/LS_Release_2.0_Access_Instructions_June2022.pdf). The Replay dataset will be freely available upon request (subject to participant consent) to yunzhe.liu.16@ucl.ac.uk. The Wakeman–Henson dataset is publicly available at OpenNeuro (https://openneuro.org/datasets/ds000117/versions/1.0.5). Source data are provided with this paper.

## Code availability

The code for all analysis code described here is publicly available via GitHub at https://github.com/OHBA-analysis/Tinda.

## References

[1] Van De Ville, D., Britz, J. & Michel, C. M. EEG microstate sequences in healthy humans at rest reveal scale-free dynamics. *Proc. Natl Acad. Sci. USA* **107**, 18179–18184 (2010). 20921381 10.1073/pnas.1007841107 PMC2964192

[2] Smith, S. M. et al. Correspondence of the brain’s functional architecture during activation and rest. *Proc. Natl Acad. Sci. USA* **106**, 13040–13045 (2009). 19620724 10.1073/pnas.0905267106 PMC2722273

[3] Calhoun, V. D., Kiehl, K. A. & Pearlson, G. D. Modulation of temporally coherent brain networks estimated using ICA at rest and during cognitive tasks. *Hum. Brain Mapp.* **29**, 828–838 (2008). 18438867 10.1002/hbm.20581 PMC2649823

[4] Seeley, W. W. et al. Dissociable intrinsic connectivity networks for salience processing and executive control. *J. Neurosci.* **27**, 2349–2356 (2007). 17329432 10.1523/JNEUROSCI.5587-06.2007 PMC2680293

[5] Fox, M. D. et al. The human brain is intrinsically organized into dynamic, anticorrelated functional networks. *Proc. Natl Acad. Sci. USA* **102**, 9673–9678 (2005). 15976020 10.1073/pnas.0504136102 PMC1157105

[6] Hutchison, R. M. et al. Dynamic functional connectivity: promise, issues, and interpretations. *NeuroImage* **80**, 360–378 (2013). 23707587 10.1016/j.neuroimage.2013.05.079 PMC3807588

[7] Calhoun, A. J., Pillow, J. W. & Murthy, M. Unsupervised identification of the internal states that shape natural behavior. *Nat. Neurosci.* **22**, 2040–2049 (2019). 31768056 10.1038/s41593-019-0533-x PMC7819718

[8] Cole, M. W., Bassett, D. S., Power, J. D., Braver, T. S. & Petersen, S. E. Intrinsic and task-evoked network architectures of the human brain. *Neuron* **83**, 238–251 (2014). 24991964 10.1016/j.neuron.2014.05.014 PMC4082806

[9] Haimovici, A., Tagliazucchi, E., Balenzuela, P. & Chialvo, D. R. Brain organization into resting state networks emerges at criticality on a model of the human connectome. *Phys. Rev. Lett.* **110**, 178101 (2013). 23679783 10.1103/PhysRevLett.110.178101

[10] Lehmann, D., Ozaki, H. & Pal, I. EEG alpha map series: brain micro-states by space-oriented adaptive segmentation. *Electroencephal. Clin. Neurophysiol*. **67**, 271–288 (1987). 10.1016/0013-4694(87)90025-3 2441961

[11] Baker, A. P. et al. Fast transient networks in spontaneous human brain activity. *eLife* **2014**, 1–18 (2014). 10.7554/eLife.01867 PMC3965210 24668169

[12] Hillebrand, A. et al. Direction of information flow in large-scale resting-state networks is frequency-dependent. *Proc. Natl Acad. Sci. USA* **113**, 3867–3872 (2016). 27001844 10.1073/pnas.1515657113 PMC4833227

[13] Sitnikova, T. A., Hughes, J. W., Ahlfors, S. P., Woolrich, M. W. & Salat, D. H. Short timescale abnormalities in the states of spontaneous synchrony in the functional neural networks in Alzheimer’s disease. *Neuroimage Clin.* **20**, 128–152 (2018). 30094163 10.1016/j.nicl.2018.05.028 PMC6077178

[14] Vidaurre, D., Smith, S. M. & Woolrich, M. W. Brain network dynamics are hierarchically organized in time. *Proc. Natl Acad. Sci. USA* **114**, 12827–12832 (2017). 29087305 10.1073/pnas.1705120114 PMC5715736

[15] Razi, A. et al. Large-scale DCMs for resting-state fMRI. *Netw. Neurosci.* **1**, 222–241 (2017). 29400357 10.1162/NETN_a_00015 PMC5796644

[16] Cabral, J. et al. Cognitive performance in healthy older adults relates to spontaneous switching between states of functional connectivity during rest. *Sci. Rep.* **7**, 5135 (2017). 28698644 10.1038/s41598-017-05425-7 PMC5506029

[17] de Pasquale, F. et al. Temporal dynamics of spontaneous MEG activity in brain networks. *Proc. Natl Acad. Sci. USA* **107**, 6040–6045 (2010). 20304792 10.1073/pnas.0913863107 PMC2851876

[18] Vidaurre, D. et al. Discovering dynamic brain networks from big data in rest and task. *NeuroImage* **180**, 646–656 (2018). 28669905 10.1016/j.neuroimage.2017.06.077 PMC6138951

[19] Deco, G., Sanz Perl, Y., Bocaccio, H., Tagliazucchi, E. & Kringelbach, M. L. The INSIDEOUT framework provides precise signatures of the balance of intrinsic and extrinsic dynamics in brain states. *Commun. Biol.* **5**, 572 (2022). 35688893 10.1038/s42003-022-03505-7 PMC9187708

[20] G-Guzmán, E. et al. The lack of temporal brain dynamics asymmetry as a signature of impaired consciousness states. *Interface Focus* **13**, 20220086 (2023). 37065259 10.1098/rsfs.2022.0086 PMC10102727

[21] Sanz Perl, Y. et al. Nonequilibrium brain dynamics as a signature of consciousness. *Phys. Rev. E* **104**, 014411 (2021). 34412335 10.1103/PhysRevE.104.014411

[22] Lynn, C. W., Cornblath, E. J., Papadopoulos, L., Bertolero, M. A. & Bassett, D. S. Broken detailed balance and entropy production in the human brain. *Proc. Natl Acad. Sci. USA* **118**, e2109889118 (2021). 34789565 10.1073/pnas.2109889118 PMC8617485

[23] Deco, G. et al. The arrow of time of brain signals in cognition: potential intriguing role of parts of the default mode network. *Netw. Neurosci.* **7**, 966–998 (2023). 37781151 10.1162/netn_a_00300 PMC10473271

[24] Higgins, C. et al. Replay bursts in humans coincide with activation of the default mode and parietal alpha networks. *Neuron* **109**, 882–893.e7 (2021). 33357412 10.1016/j.neuron.2020.12.007 PMC7927915

[25] MEG UK Database (UK MEG Partnership, 2016); https://meguk.ac.uk/database/

[26] Cam-CAN et al. The Cambridge Centre for Ageing and Neuroscience (Cam-CAN) study protocol: a cross-sectional, lifespan, multidisciplinary examination of healthy cognitive ageing. *BMC Neurol.* **14**, 204 (2014). 25412575 10.1186/s12883-014-0204-1 PMC4219118

[27] Taylor, J. R. et al. The Cambridge Centre for Ageing and Neuroscience (Cam-CAN) data repository: structural and functional MRI, MEG, and cognitive data from a cross-sectional adult lifespan sample. *NeuroImage* **144**, 262–269 (2017). 26375206 10.1016/j.neuroimage.2015.09.018 PMC5182075

[28] Larson-Prior, L. J. et al. Adding dynamics to the Human Connectome Project with MEG. *NeuroImage* **80**, 190–201 (2013). 23702419 10.1016/j.neuroimage.2013.05.056 PMC3784249

[29] Vidaurre, D. et al. Spontaneous cortical activity transiently organises into frequency specific phase-coupling networks. *Nat. Commun.* **9**, 2987 (2018). 30061566 10.1038/s41467-018-05316-z PMC6065434

[30] Mitra, A. & Raichle, M. E. How networks communicate: propagation patterns in spontaneous brain activity. *Philos. Trans. R. Soc. B* **371**, 20150546 (2016). 10.1098/rstb.2015.0546 PMC5003863 27574315

[31] Thomas Yeo, B. T. et al. The organization of the human cerebral cortex estimated by intrinsic functional connectivity. *J. Neurophysiol.* **106**, 1125–1165 (2011). 21653723 10.1152/jn.00338.2011 PMC3174820

[32] Sepulcre, J., Sabuncu, M. R., Yeo, T. B., Liu, H. & Johnson, K. A. Stepwise connectivity of the modal cortex reveals the multimodal organization of the human brain. *J. Neurosci.* **32**, 10649–10661 (2012). 22855814 10.1523/JNEUROSCI.0759-12.2012 PMC3483645

[33] Smith, S. M. et al. A positive-negative mode of population covariation links brain connectivity, demographics and behavior. *Nat. Neurosci.* **18**, 1565–1567 (2015). 26414616 10.1038/nn.4125 PMC4625579

[34] Chen, X., Viding, E. & Nichols, T. E. Faster Accelerated Permutation Inference for the ACE Model (APACE) with parallelization. *figshare*10.6084/M9.FIGSHARE.5473174.V1 (2017).

[35] Eaves, L. et al. Comparing the biological and cultural inheritance of personality and social attitudes in the Virginia 30,000 study of twins and their relatives. *Twin Res.* **2**, 62–80 (1999). 10480741 10.1375/136905299320565933

[36] Liu, Y., Dolan, R. J., Kurth-Nelson, Z. & Behrens, T. E. J. Human replay spontaneously reorganizes experience. *Cell* **178**, 640–652.e14 (2019). 31280961 10.1016/j.cell.2019.06.012 PMC6657653

[37] Wakeman, D. G. & Henson, R. N. A multi-subject, multi-modal human neuroimaging dataset. *Sci. Data* **2**, 150001 (2015). 25977808 10.1038/sdata.2015.1 PMC4412149

[38] Quinn, A. J. et al. Task-evoked dynamic network analysis through hidden markov modeling. *Front. Neurosci.* **12**, 603 (2018). 30210284 10.3389/fnins.2018.00603 PMC6121015

[39] Bijsterbosch, J. et al. Investigations into within- and between-subject resting-state amplitude variations. *NeuroImage* **159**, 57–69 (2017). 28712995 10.1016/j.neuroimage.2017.07.014 PMC5678294

[40] Doucet, G. et al. Brain activity at rest: a multiscale hierarchical functional organization. *J. Neurophysiol.* **105**, 2753–2763 (2011). 21430278 10.1152/jn.00895.2010

[41] Margulies, D. S. et al. Situating the default-mode network along a principal gradient of macroscale cortical organization. *Proc. Natl Acad. Sci. USA* **113**, 12574–12579 (2016). 27791099 10.1073/pnas.1608282113 PMC5098630

[42] Gnesotto, F. S., Mura, F., Gladrow, J. & Broedersz, C. P. Broken detailed balance and non-equilibrium dynamics in living systems: a review. *Rep. Prog. Phys.* **81**, 066601 (2018). 29504517 10.1088/1361-6633/aab3ed

[43] Cruzat, J. et al. Temporal irreversibility of large-scale brain dynamics in alzheimer’s disease. *J. Neurosci.* **43**, 1643–1656 (2023). 36732071 10.1523/JNEUROSCI.1312-22.2022 PMC10008060

[44] Sporns, O. & Kötter, R. Motifs in brain networks. *PLoS Biol.* **2**, e369 (2004). 15510229 10.1371/journal.pbio.0020369 PMC524253

[45] Milo, R. et al. Network motifs: simple building blocks of complex networks. *Science*10.1126/science.298.5594.824 (2002). 10.1126/science.298.5594.824 12399590

[46] Felleman, D. J. & Van Essen, D. C. Distributed hierarchical processing in the primate cerebral cortex. *Cereb. Cortex* **1**, 1–47 (1991). 1822724 10.1093/cercor/1.1.1-a

[47] Deco, G., Cruzat, J. & Kringelbach, M. L. Brain songs framework used for discovering the relevant timescale of the human brain. *Nat. Commun.* **10**, 583 (2019). 30718478 10.1038/s41467-018-08186-7 PMC6361902

[48] Kobeleva, X., López-González, A., Kringelbach, M. L. & Deco, G. Revealing the relevant spatiotemporal scale underlying whole-brain dynamics. *Front. Neurosci*. **15**, 715861 (2021). 10.3389/fnins.2021.715861 PMC8569182 34744605

[49] Magnusson, K. R. & Brim, B. L. The Aging Brain in *Reference Module in Biomedical Sciences* (ed. Caplan, M. J.) (Elsevier, 2014).

[50] Merkin, A. et al. Do age-related differences in aperiodic neural activity explain differences in resting EEG alpha? *Neurobiol. Aging* **121**, 78–87 (2023). 36379095 10.1016/j.neurobiolaging.2022.09.003

[51] Vlahou, E. L., Thurm, F., Kolassa, I.-T. & Schlee, W. Resting-state slow wave power, healthy aging and cognitive performance. *Sci. Rep.* **4**, 5101 (2014). 24869503 10.1038/srep05101 PMC4037748

[52] Scally, B., Burke, M. R., Bunce, D. & Delvenne, J.-F. Resting-state EEG power and connectivity are associated with alpha peak frequency slowing in healthy aging. *Neurobiol. Aging* **71**, 149–155 (2018). 30144647 10.1016/j.neurobiolaging.2018.07.004

[53] Chen, Q. et al. Decreased inter-hemispheric interactions but increased intra-hemispheric integration during typical aging. *Aging* **11**, 10100–10115 (2019). 31761785 10.18632/aging.102421 PMC6914428

[54] Geerligs, L., Renken, R. J., Saliasi, E., Maurits, N. M. & Lorist, M. M. A brain-wide study of age-related changes in functional connectivity. *Cereb. Cortex* **25**, 1987–1999 (2015). 24532319 10.1093/cercor/bhu012

[55] He, L., Wang, X., Zhuang, K. & Qiu, J. Decreased dynamic segregation but increased dynamic integration of the resting-state functional networks during normal aging. *Neuroscience* **437**, 54–63 (2020). 32353459 10.1016/j.neuroscience.2020.04.030

[56] King, B. R. et al. Age-related declines in motor performance are associated with decreased segregation of large-scale resting state brain networks. *Cereb. Cortex* **28**, 4390–4402 (2018). 29136114 10.1093/cercor/bhx297 PMC6215458

[57] Schlesinger, K. J., Turner, B. O., Lopez, B. A., Miller, M. B. & Carlson, J. M. Age-dependent changes in task-based modular organization of the human brain. *NeuroImage* **146**, 741–762 (2017). 27596025 10.1016/j.neuroimage.2016.09.001

[58] Adhikari, B. M. et al. Comparison of heritability estimates on resting state fMRI connectivity phenotypes using the ENIGMA analysis pipeline. *Hum. Brain Mapp.* **39**, 4893–4902 (2018). 30052318 10.1002/hbm.24331 PMC6218292

[59] Barber, A. D., Hegarty, C. E., Lindquist, M. & Karlsgodt, K. H. Heritability of functional connectivity in resting state: assessment of the dynamic mean, dynamic variance, and static connectivity across networks. *Cereb. Cortex* **31**, 2834–2844 (2021). 33429433 10.1093/cercor/bhaa391 PMC8325018

[60] Fu, Y. et al. Genetic influences on resting-state functional networks: a twin study: genetic influences on resting-state functional networks. *Hum. Brain Mapp.* **36**, 3959–3972 (2015). 26147340 10.1002/hbm.22890 PMC4704468

[61] Glahn, D. C. et al. Genetic control over the resting brain. *Proc. Natl Acad. Sci. USA* **107**, 1223–1228 (2010). 20133824 10.1073/pnas.0909969107 PMC2824276

[62] Schutte, N. M. et al. Heritability of resting state EEG functional connectivity patterns. *Twin Res. Hum. Genet.* **16**, 962–969 (2013). 23931641 10.1017/thg.2013.55

[63] Smit, D. J. A., Stam, C. J., Posthuma, D., Boomsma, D. I. & De Geus, E. J. C. Heritability of ‘small-world’ networks in the brain: a graph theoretical analysis of resting-state EEG functional connectivity. *Hum. Brain Mapp.* **29**, 1368–1378 (2008). 18064590 10.1002/hbm.20468 PMC6870849

[64] Morowitz, H. J. Physical background of cycles in biological systems. *J. Theor. Biol.* **13**, 60–62 (1966).

[65] Loomis, A. L., Harvey, E. N. & Hobart, G. A. Cerebral states during sleep, as studied by human brain potentials. *J. Exp. Psychol.* **21**, 127–144 (1937).

[66] Gohil, C. et al. Mixtures of large-scale dynamic functional brain network modes. *NeuroImage* **263**, 119595 (2022). 36041643 10.1016/j.neuroimage.2022.119595 PMC7618940

[67] Uddin, L. Q. et al. Controversies and progress on standardization of large-scale brain network nomenclature. *Netw. Neurosci.* **7**, 864–905 (2023). 37781138 10.1162/netn_a_00323 PMC10473266

[68] Kong, R. et al. A network correspondence toolbox for quantitative evaluation of novel neuroimaging results. *Nat. Commun.* **16**, 2930 (2025). 40133295 10.1038/s41467-025-58176-9 PMC11937327

[69] Gohil, C. et al. osl-dynamics, a toolbox for modeling fast dynamic brain activity. *eLife* **12**, RP91949 (2024). 38285016 10.7554/eLife.91949 PMC10945565

[70] Sitnikova, T. et al. Spontaneous activity changes in large-scale cortical networks in older adults couple to distinct hemodynamic morphology. Preprint at *bioRxiv* http://biorxiv.org/lookup/doi/10.1101/2020.05.05.079749 (2020).

[71] Cho, S., Van Es, M., Woolrich, M. & Gohil, C. Comparison between EEG and MEG of static and dynamic resting‐state networks. *Hum. Brain Mapp.* **45**, e70018 (2024). 39230193 10.1002/hbm.70018 PMC11372824

[72] Mantini, D., Perrucci, M. G., Del Gratta, C., Romani, G. L. & Corbetta, M. Electrophysiological signatures of resting state networks in the human brain. *Proc. Natl Acad. Sci. USA* **104**, 13170–13175 (2007). 17670949 10.1073/pnas.0700668104 PMC1941820

[73] Laufs, H. et al. EEG-correlated fMRI of human alpha activity. *NeuroImage* **19**, 1463–1476 (2003). 12948703 10.1016/s1053-8119(03)00286-6

[74] Knyazev, G. G., Slobodskoj-Plusnin, J. Y., Bocharov, A. V. & Pylkova, L. V. The default mode network and EEG alpha oscillations: an independent component analysis. *Brain Res.* **1402**, 67–79 (2011). 21683942 10.1016/j.brainres.2011.05.052

[75] Marino, M., Arcara, G., Porcaro, C. & Mantini, D. Hemodynamic correlates of electrophysiological activity in the default mode network. *Front. Neurosci.* **13**, 1060 (2019). 31636535 10.3389/fnins.2019.01060 PMC6788217

[76] Jann, K. et al. BOLD correlates of EEG alpha phase-locking and the fMRI default mode network. *NeuroImage* **45**, 903–916 (2009). 19280706 10.1016/j.neuroimage.2009.01.001

[77] MATLAB (The MathWorks Inc., 2020).

[78] OSL MATLAB (OHBA Analysis Group, 2014).

[79] Quinn, A. J., van Es, M. W. J., Gohil, C. & Woolrich, M. W. OHBA software library in Python (OSL). *Zenodo*10.5281/ZENODO.6875060 (2022).

[80] Van Es, M. W. J., Gohil, C., Quinn, A. J. & Woolrich, M. W. osl-ephys: a Python toolbox for the analysis of electrophysiology data. *Front. Neurosci.* **19**, 1522675 (2025). 40061258 10.3389/fnins.2025.1522675 PMC11885225

[81] HMM-MAR (OHBA Analysis Group, 2016).

[82] Gramfort, A. MEG and EEG data analysis with MNE-Python. *Front. Neurosci.* **7**, 267 (2013). 24431986 10.3389/fnins.2013.00267 PMC3872725

[83] Larson, E. et al. MNE-Python. *Zenodo*10.5281/ZENODO.592483 (2023).

[84] van Es, M.W.J. & Higgins, C. TINDA (2023).

[85] Hunt, B. A. E. et al. Relationships between cortical myeloarchitecture and electrophysiological networks. *Proc. Natl Acad. Sci. USA* **113**, 13510–13515 (2016). 27830650 10.1073/pnas.1608587113 PMC5127325

[86] Tzourio-Mazoyer, N. et al. Automated anatomical labeling of activations in SPM using a macroscopic anatomical parcellation of the MNI MRI single-subject brain. *NeuroImage* **15**, 273–289 (2002). 11771995 10.1006/nimg.2001.0978

[87] Colclough, G. L., Brookes, M. J., Smith, S. M. & Woolrich, M. W. A symmetric multivariate leakage correction for MEG connectomes. *NeuroImage* **117**, 439–448 (2015). 25862259 10.1016/j.neuroimage.2015.03.071 PMC4528074

[88] Duff, I. S. & Koster, J. On algorithms for permuting large entries to the diagonal of a sparse matrix. *SIAM J. Matrix Anal. Appl.* **22**, 973–996 (2001).

[89] Higgins, C. et al. Spatiotemporally resolved multivariate pattern analysis for M/EEG. *Hum. Brain Mapp.*10.1002/hbm.25835 (2022). 10.1002/hbm.25835 PMC9188977 35302683

[90] Salarian, A. Intraclass Correlation Coefficient (ICC). *MATLAB Central File Exchange* https://www.mathworks.com/matlabcentral/fileexchange/22099-intraclass-correlation-coefficient-icc (2024).

[91] Chen, X. et al. Accelerated estimation and permutation inference for ACE modeling. *Hum. Brain Mapp.* **40**, 3488–3507 (2019). 31037793 10.1002/hbm.24611 PMC6680147

[92] Fischl, B. FreeSurfer. *NeuroImage* **62**, 774–781 (2012). 22248573 10.1016/j.neuroimage.2012.01.021 PMC3685476

