# Supplementary material for: Large-scale cortical functional networks are organized in structured cycles
Source: Nat Neurosci. 2025 Aug 27;28(10):2118–28. doi: 10.1038/s41593-025-02052-8 (PMC12497652; doi:10.1038/s41593-025-02052-8)
Supplement: Supplementary file 2 — Reporting Summary [file 41593_2025_2052_MOESM2_ESM.pdf]

nature portfolio

Corresponding author(s): Mats W.J. van Es
Last updated by author(s): Jun 11, 2025

Reporting Summary

Nature Portfolio wishes to improve the reproducibility of the work that we publish. This form provides structure for consistency and transparency in reporting. For further information on Nature Portfolio policies, see our Editorial Policies and the Editorial Policy Checklist.

Statistics

For all statistical analyses, confirm that the following items are present in the figure legend, table legend, main text, or Methods section.

n/a	Confirmed	
☐	☒	The exact sample size (*n*) for each experimental group/condition, given as a discrete number and unit of measurement
☐	☒	A statement on whether measurements were taken from distinct samples or whether the same sample was measured repeatedly
☐	☒	The statistical test(s) used AND whether they are one- or two-sided<br>*Only common tests should be described solely by name; describe more complex techniques in the Methods section.*
☐	☒	A description of all covariates tested
☐	☒	A description of any assumptions or corrections, such as tests of normality and adjustment for multiple comparisons
☐	☒	A full description of the statistical parameters including central tendency (e.g. means) or other basic estimates (e.g. regression coefficient) AND variation (e.g. standard deviation) or associated estimates of uncertainty (e.g. confidence intervals)
☐	☒	For null hypothesis testing, the test statistic (e.g. F, t, r) with confidence intervals, effect sizes, degrees of freedom and P value noted<br>*Give P values as exact values whenever suitable.*
☒	☐	For Bayesian analysis, information on the choice of priors and Markov chain Monte Carlo settings
☒	☐	For hierarchical and complex designs, identification of the appropriate level for tests and full reporting of outcomes
☐	☒	Estimates of effect sizes (e.g. Cohen's *d*, Pearson's *r*), indicating how they were calculated

Our web collection on statistics for biologists contains articles on many of the points above.

Software and code

Policy information about availability of computer code

Data collection	No software was used for data collection.
Data analysis	All data were analysed using MATLAB 2023a, Python 3.8, FSL 6.0.5, and custom-written scripts (available at https://github.com/OHBA-analysis/Tinda). In addition, the following Python/MATLAB based toolboxes were used: OHBA Software Library (https://github.com/OHBA-analysis/osl-core), HMM-MAR (https://github.com/OHBA-analysis/HMM-MAR/), OSL-Dynamics (https://github.com/OHBA-analysis/osl-dynamics), OSL-Ephys (https://github.com/OHBA-analysis/osl-ephys/tree/main), and MNE-Python (https://mne.tools/stable/index.html)

For manuscripts utilizing custom algorithms or software that are central to the research but not yet described in published literature, software must be made available to editors and reviewers. We strongly encourage code deposition in a community repository (e.g. GitHub). See the Nature Portfolio guidelines for submitting code & software for further information.

Data

Policy information about availability of data

All manuscripts must include a data availability statement. This statement should provide the following information, where applicable:

- Accession codes, unique identifiers, or web links for publicly available datasets
- A description of any restrictions on data availability
- For clinical datasets or third party data, please ensure that the statement adheres to our policy

All data used for this manuscript are third-party data, which are available at the respective databases. The MEG UK Partnership data is held by the MEG UK Partnership, for which access can be requested at https://meguk.ac.uk/database/. The Cam-CAN dataset is available upon request to https://camcan-archive.mrc-cbu.cam.ac.uk/dataaccess/datarequest.php. The HCP dataset is freely available at https://db.humanconnectome.org/app/template/Login.vm but will require an application for sensitive data (see https://www.humanconnectome.org/storage/app/media/documentation/LS2.0/LS_Release_2.0_Access_Instructions_June2022.pdf). The Replay dataset will be freely available upon request (subject to participant consent) to yunzhe.liu.16@ucl.ac.uk. The Wakeman-Henson dataset is publicaly available at OpenNeuro (https://openneuro.org/datasets/ds000117/versions/1.0.5).

Research involving human participants, their data, or biological material

Policy information about studies with human participants or human data. See also policy information about sex, gender (identity/presentation), and sexual orientation and race, ethnicity and racism.

Reporting on sex and gender	Only reporting on sex, as gender was not collected
Reporting on race, ethnicity, or other socially relevant groupings	Data not available
Population characteristics	MEG UK (N=55): mean age 37.8 (range 19-62), 26 male/29 female<br>Cam-CAM: mean age 54.6 (range 18-88), 310 male/302 female<br>HCP: mean age 39 (range 22-35), 42 male/37 female, 13 mono- and 11-dizygotic twin pairs<br>Replay dataset1: mean age 24.9 (range 19-34), 11 male/14 female<br>Replay dataset2: mean age 25.5 (range 19-34), 10 male/16 female<br>Wakeman-Henson: age range 23-37, 11 male/8 female
Recruitment	See original data publications
Ethics oversight	No ethical approval was required for the analysis of the public data. The ethical approvals for each of the datasets were acquired by the respective study teams, as outlined below:<br>MEG UK: ethical approval was granted by the University of Nottingham Medical School Research Ethics Committee.<br>Cam-CAN: ethical approval was granted by the Cambridgeshire Research Ethics Committee<br>HCP: ethical approval was granted by the local ethics committee.<br>Replay dataset1: ethical approval for the experiment was obtained from the Research Ethics Committee at University College London under ethics number 9929/002<br>Replay dataset2: ethical approval for the experiment was obtained from the Research Ethics Committee at University College London under ethics number 9929/002<br>Wakeman-Henson: ethical approval was obtained from the Cambridge University Psychological Ethics Committee

Note that full information on the approval of the study protocol must also be provided in the manuscript.

Field-specific reporting

Please select the one below that is the best fit for your research. If you are not sure, read the appropriate sections before making your selection.

☒ Life sciences ☐ Behavioural & social sciences ☐ Ecological, evolutionary & environmental sciences

For a reference copy of the document with all sections, see nature.com/documents/nr-reporting-summary-flat.pdf

Life sciences study design

All studies must disclose on these points even when the disclosure is negative.

Sample size	Sample sizes were limited by the availability of public datasets, and were not predetermined based on power calculations.<br><br>Dataset 1 (MEG UK), N=55<br>Dataset 2 (Cam-CAN), N=612<br>Dataset 3 (HCP), N=79<br>Dataset 4 (Replay), N=43<br>Dataset 5 (Wakeman-Henson), N=19

Data exclusions	MEG UK: 22 subjects were excluded from the original dataset (N=77) because of excessive head movements or artifacts in the data.<br>Cam-CAN: 41 subjects were excluded from the original dataset (N=653) because of incompleteness of the data<br>HCP: 21 subjects were excluded from the original dataset (N=100) because of excessive noise.<br>Replay dataset1: 4 subjects were excluded from the original dataset (N=25) because of large motion artifacts or missing trigger information<br>Replay dataset2: 4 subjects were excluded from the original dataset (N=26) because of large motion artifacts or failure to compete the task.
Replication	Core results were replicated in five independent dataset, all of which were successful.
Randomization	Subjects were not allocated to a specific group - existing allocations were used based on the original study designs.
Blinding	Blinding was not relevant to this study because of the absence of a priori hypotheses regarding participant demographics. Furthermore, the availability of public datasets limited blinding.

Reporting for specific materials, systems and methods

We require information from authors about some types of materials, experimental systems and methods used in many studies. Here, indicate whether each material, system or method listed is relevant to your study. If you are not sure if a list item applies to your research, read the appropriate section before selecting a response.

Materials & experimental systems

n/a	Involved in the study
☒	☐ Antibodies
☒	☐ Eukaryotic cell lines
☒	☐ Palaeontology and archaeology
☒	☐ Animals and other organisms
☒	☐ Clinical data
☒	☐ Dual use research of concern
☒	☐ Plants

Methods

n/a	Involved in the study
☒	☐ ChIP-seq
☒	☐ Flow cytometry
☐	☒ MRI-based neuroimaging

Plants

Seed stocks	*Report on the source of all seed stocks or other plant material used. If applicable, state the seed stock centre and catalogue number. If plant specimens were collected from the field, describe the collection location, date and sampling procedures.*
Novel plant genotypes	*Describe the methods by which all novel plant genotypes were produced. This includes those generated by transgenic approaches, gene editing, chemical/radiation-based mutagenesis and hybridization. For transgenic lines, describe the transformation method, the number of independent lines analyzed and the generation upon which experiments were performed. For gene-edited lines, describe the editor used, the endogenous sequence targeted for editing, the targeting guide RNA sequence (if applicable) and how the editor was applied.*
Authentication	*Describe any authentication procedures for each seed stock used or novel genotype generated. Describe any experiments used to assess the effect of a mutation and, where applicable, how potential secondary effects (e.g. second site T-DNA insertions, mosiacism, off-target gene editing) were examined.*

Magnetic resonance imaging

Experimental design

Design type	*Indicate task or resting state; event-related or block design.*
Design specifications	*Specify the number of blocks, trials or experimental units per session and/or subject, and specify the length of each trial or block (if trials are blocked) and interval between trials.*
Behavioral performance measures	*State number and/or type of variables recorded (e.g. correct button press, response time) and what statistics were used to establish that the subjects were performing the task as expected (e.g. mean, range, and/or standard deviation across subjects).*

Acquisition

Imaging type(s) | Structural

Field strength | dataset1: 3T
dataset 2: 3T
dataset 3: 3T
dataset 5: 3T

Sequence & imaging parameters | dataset 1: 1 × 1 × 1 mm3 resolution running an MPRAGE sequence
dataset 2: Prepared RApid Gradient Echo (MPRAGE) sequence with the following parameters: Repetition Time (TR) =2250 milleseconds; Echo Time (TE) =2.99 milliseconds; Inversion Time (TI) =900 milliseconds; flip angle =9 degrees; field of view (FOV) =256mm x 240mm x 192mm; voxel size =1mm isotropic; GRAPPA acceleration factor =2; acquisition time of 4 minutes and 32 seconds.
dataset 3: MPRAGE; TR/TE = 2400/2.14 ms, 192 slices, 0.7 mm3 isotropic resolution, TI = 1000 ms, parallel imaging (2×, GRAPPA)
dataset 5: 1 mm isotropic T1-weighted 'structural' image was acquired using an MPRAGE sequence (TR 2,250 ms, TE 2.98 ms, TI 900 ms, 190 Hz/pixel; flip angle 9°)

Area of acquisition | whole-brain

Diffusion MRI ☐ Used ☒ Not used

Preprocessing

Preprocessing software | Matlab, OSL-Matlab, OSL-ephys, OSL-dynamics, MNE-Python, FSL,

Normalization | *If data were normalized/standardized, describe the approach(es): specify linear or non-linear and define image types used for transformation OR indicate that data were not normalized and explain rationale for lack of normalization.*

Normalization template | MNI152

Noise and artifact removal | *Describe your procedure(s) for artifact and structured noise removal, specifying motion parameters, tissue signals and physiological signals (heart rate, respiration).*

Volume censoring | *Define your software and/or method and criteria for volume censoring, and state the extent of such censoring.*

Statistical modeling & inference

Model type and settings | MEG source-modelling, intracranial volume

Effect(s) tested | *Define precise effect in terms of the task or stimulus conditions instead of psychological concepts and indicate whether ANOVA or factorial designs were used.*

Specify type of analysis: ☒ Whole brain ☐ ROI-based ☐ Both

Statistic type for inference | *Specify voxel-wise or cluster-wise and report all relevant parameters for cluster-wise methods.*

(See Eklund et al. 2016)

Correction | *Describe the type of correction and how it is obtained for multiple comparisons (e.g. FWE, FDR, permutation or Monte Carlo).*

Models & analysis

n/a	Involved in the study
☒	☐ Functional and/or effective connectivity
☒	☐ Graph analysis
☐	☒ Multivariate modeling or predictive analysis

Multivariate modeling and predictive analysis | T1 scans were used for MEG source modelling
Intracranial volume was used as a confound regressor in testing heritability of cycle rate and cycle strength
